# Foveal feedback in perceptual processing: Contamination of neural representations and task difficulty effects

**DOI:** 10.1371/journal.pone.0291275

**Published:** 2023-10-05

**Authors:** Giulio Contemori, Carolina Maria Oletto, Luca Battaglini, Elena Motterle, Marco Bertamini

**Affiliations:** Department of General Psychology, University of Padova, Padova, Italy; Justus Liebig Universitat Giessen, GERMANY

## Abstract

Visual object recognition was traditionally believed to rely on a hierarchical feedforward process. However, recent evidence challenges this notion by demonstrating the crucial role of foveal retinotopic cortex and feedback signals from higher-level visual areas in processing peripheral visual information. The nature of the information conveyed through foveal feedback remains a topic of debate. To address this, we conducted a study employing a foveal mask paradigm with varying stimulus-mask onset asynchronies in a peripheral same/different task, where peripheral objects exhibited different degrees of similarity. Our hypothesis posited that simultaneous arrival of feedback and mask information in the foveal cortex would lead to neural contamination, biasing perception. Notably, when the two peripheral objects were identical, we observed a significant increase in the number of "different" responses, peaking at approximately 100 ms. Similar effect was found when the objects were dissimilar, but with an overall later timing (around 150 ms). No significant difference was found when comparing easy (dissimilar objects) and difficult trials (similar objects). The findings challenge the hypothesis that foveation planning alone accounts for the observed effects. Instead, these and previous observations support the notion that the foveal cortex serves as a visual sketchpad for maintaining and manipulating task-relevant information.

## Introduction

Traditionally, visual object recognition was thought to rely primarily on a hierarchical feedforward, with early processing stages being strongly retinotopic. Recent evidence has shown that visual processing is more flexible, and that the foveal retinotopic cortex plays an important role in processing information presented in the periphery of the visual field (for a review see Stewart et al., 2020) [1]. This is achieved through feedback signals from higher-level visual areas, which recruit the foveal cortex to participate in object recognition as an auxiliary computational module [2]. Evidence for the role of this foveal feedback in peripherally presented object discrimination has been provided by studies using transcranial magnetic stimulation (TMS): disruption of the foveal processing after, but not during, stimulus presentation affects performance [3]. Similar results have been found by disrupting foveal processing with a central mask presented at variable stimulus onset asynchrony. Despite differences in design, a performance drop is generally observed for masks presented between 100 and 300 ms after stimulus onset (for a review see Oletto et al., 2022) [4]. One possibility is that this feedback is necessary to recruit the small receptive fields in the foveal cortex as an adjunctive computational module for processing fine details in the peripheral visual field. Another hypothesis is that it is preparatory to foveation and is therefore a by-product of saccade planning [2, 5].

Although there is support in favour of this second hypothesis [6, 7], the preparation of foveation cannot account for all findings [7, 8]. First, fMRI studies have shown that the amount of information present in the foveal retinotopic cortex is positively correlated with task performance during peripheral discrimination tasks, indicating that feedback is meaningful even in the absence of later foveation [2]. Second, in behavioural paradigms, the timing at which the foveal mask causes the maximum drop in performance is not necessarily time-locked to saccade planning, and may be delayed if the task requires mental manipulation of the target [7]. Third, disrupting the feedback not only affects sensitivity but also response bias, with participants tending to be more conservative in their decision-making processes [8]. These observations suggest that the foveal mask influences the decisional process beyond the planning of the saccade.

The nature of the information fed back to the foveal sketchpad is debated. When the task is based on discrimination of low-level features [9] or blurred stimuli [7], the foveal mask produces no modulation in performance. Thus, only high-level detailed object information seems to be fed back. The finding that the foveal mask impairs category discrimination for subordinate but not supraordinate categories [10] further supports this hypothesis. Moreover, fMRI data show that foveal retinotopic cortex contains both information about category and orientation of the peripheral object [7, 9].

Another visual ability that appears to be influenced by the presence of the foveal mask is colour discrimination. A delayed foveal mask negatively affects colour discrimination when target and mask were coloured, but not when mask was greyscale. On the contrary, when the task was about shape, both coloured and grayscale mask produced a similar disruptive effect [11]. This suggests that the mask is more effective when it shares some of the task-related properties of the stimulus. This is corroborated by two other studies were the object acting as mask could be congruent or incongruent with respect to the target. Results show increased performance for congruent and decreased performance for incongruent masks [6, 9]. This pooling of features between peripheral target and foveal mask could also explain the conservative shift in the criterion found by Contemori et al. in their recent work [8].

Within the framework of Signal Detection Theory, the term "Criterion" (C), or "bias", denotes the individual decision threshold or standard of judgment applied to categorize sensory stimuli as either signal or noise. In their investigation, Contemori et al. [8] found that participants adopted a more conservative criterion when the stimulus was masked. The peak of the criterion shift was observed approximately with 177 ms delay between target and mask. These properties are not fully explained by a predictive mechanism for foveation, and may be better accounted for by thinking of the foveal retinotopic cortex as a visual sketchpad for the maintenance and manipulation of task-relevant information, similar to Baddeley’s visuospatial sketchpad (VSSP) [4].

The prevailing theory suggests that foveal feedback serves to recruit the fine-grained spatial resolution neurons of the foveal cortex to process extra-foveal shape information [2, 3]. This mechanism likely aims to increase the precision of perceptual decisions [12–14]. The co-occurrence of foveal noise during the reconstruction of peripheral stimuli disrupts this mechanism, causing a shift in the decision criterion [4, 8]. This account could also explain cases where congruence between peripheral stimuli and foveal foil improves performance rather than acting as masking [6, 9]. This top-down process might be a component of a larger circuit related to visual working memory [15, 16] and/or mental imagery [17, 18].

In this study, we conduct an experiment to investigate the impact of stimulus similarity and the onset asynchrony between the peripheral target and the foveal mask on the decision-making process. We focus on a peripheral same/different task where peripheral objects display varying degrees of similarity between couples. We hypothesize that when evaluating the similarity between two peripheral targets, the concurrent arrival of task-related feedback and mask’s feedforward leads to contamination of the neural representation of the targets.

Recognition of objects in the peripheral visual field is susceptible to a phenomenon known as visual crowding, wherein the presence of similar objects surrounding the target object hinders its recognition [19]. Information about the target identity can affect other tasks, despite the fact that participants are unable to identify this target [20]. The brain is predisposed to efficiently process summary information about groups of visual objects across various levels of complexity [21, 22]. Spatial proximity between the target and distracting elements is a fundamental characteristic of visual crowding [19]. In the case of foveal feedback, where the targets and the mask are situated at a considerable distance from each other, there is a possibility that temporal proximity could still lead to pooling. Interestingly, research has demonstrated that even in classical crowding, the masking effect of flanker elements increases when they are presented with a slight delay [23]. In other words, at a timing consistent with foveal feedback, we expect pooling of visual information from mask and target. This may result in a perceptual bias towards perceiving the targets as different. To investigate this hypothesis, we analysed the number of “different” responses elicited as a function of the Stimulus Onset Asynchrony (SOA) and objects similarity. Based on the criterion shift found by Contemori et al. [8], we expect the number of “different” responses to increase for SOAs between 100 and 200 ms. We also expect the number of “different” responses to change according to the similarity. We anticipate that the "same" condition will be affected earlier than the "different" condition, providing an explanation for the discrepancy in timing between the dip in discriminability (d’) and the peak in criterion (C) observed in a previous study by Contemori et al. [8].

There is a second hypothesis we aim to investigate. Using a similar same/different paradigm, Fan et al. found that if one of the two peripheral targets is rotated, the delay at which the mask has the greatest disruptive effect increases as a function of the degree of rotation [7]. This effect can be interpreted in two ways: either as a consequence of the increase in task difficulty or as a consequence of the increase in task complexity, which refers to the additional mental operation required before object comparison. Interestingly, Fan et al. observed that the drop in d’ (a measure of discriminability) caused by the presence of the mask remains consistent regardless of mental rotation, while only the timing of mask effectiveness varies based on the angle of mental rotation. They also noted that the levels of task difficulty for the conditions with 40° and 80° of mental rotation were no different, as participants performed similarly in these two conditions (d′ = 1.17, d′ = 1.13; p = 0.721). This led Fan et al. to dismiss the first interpretation. Instead, they argued that only the timing of the mask’s effectiveness changes with the angle of mental rotation. This dissociation between discriminability and mask timing supports the notion that the shift in timing is due to increased task complexity rather than task difficulty.

However, it is important to note that in the same study, the performance with the original task (without mental rotation) was higher than in the one involving mental rotation. This indicates that task difficulty and task complexity were at least partially confounded, as the decline in baseline performance suggests that the addition of mental rotation could have increased task difficulty. Hence, we aim to examine the effect of task difficulty without any additional mental operations. In our study, the similarity within pairs of objects serves as a proxy for task difficulty, with less similarity between objects expected to result in easier discrimination [24]. In this context, the absence of an interaction between task difficulty and mask timing would lend further support to Fan et al.’s interpretation [7].

## Methods

**Fig 1:**
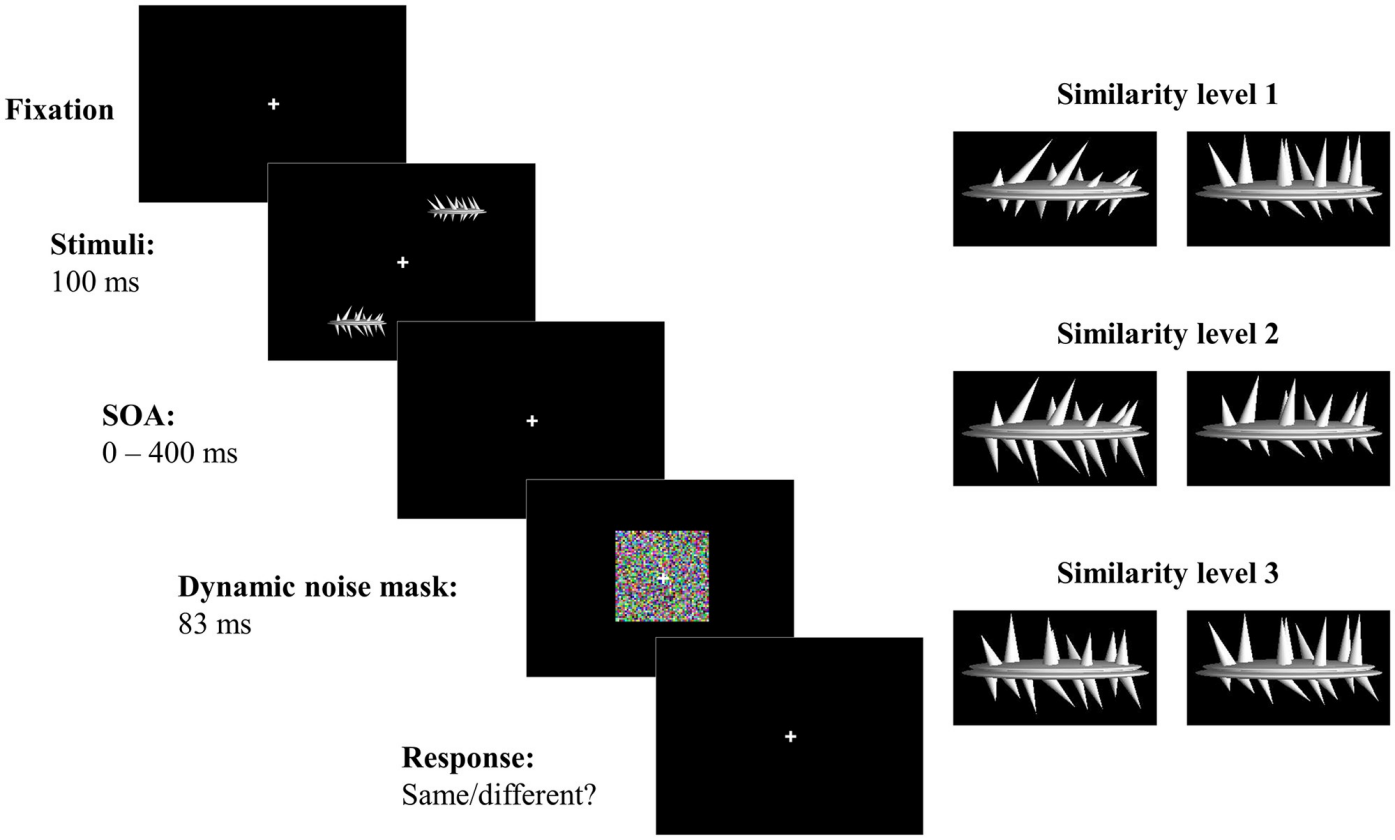
Schematic of a trial in the experiment. Two spikies were presented for 100 ms in the periphery of the visual field, in diagonally opposite quadrants. The objects differed from each other based on four primary characteristics, the length and orientation of both upper and lower spikes. A dynamic noise mask appeared in the fovea for 83 ms, introduced at one of eight possible SOAs: 50, 100, 150, 200, 250, 300, 350, and 400 ms. In the baseline condition, no noise mask was presented. Spikies pairs exemplify average similarity across three similarity levels. Level 1 represents highly dissimilar object pairs with a Jaccard coefficient of 0.513. Level 2 represents differing object pairs with a Jaccard coefficient of 0.609. Level 3 exemplifies highly similar object pairs with an average Jaccard coefficient of 0.755.

The experimental design was adapted from a previous online experiment conducted in our laboratory [8], which itself was based on the original Experiment 1 by Fan et al. [7]. Participants engaged in a same-different task involving two peripheral stimuli, either accompanied by a central dynamic coloured mask or not. The mask appeared with varying onset asynchronies in relation to the target stimuli. Target objects were abstract 3D shapes of the spiky category used by Fan et al. which have been provided to us courtesy of the authors of the original study (Fig 1). In our previous study, we examined five SOAs set at 50, 150, 250, 350, and 450 ms. However, in this study, the asynchrony between target and mask was set to 50 ms intervals ranging from 0 to 400 ms. This was done to obtain a higher SOA density while keeping the total duration of the experiment within the hour. The study consisted of a total of 40 conditions in a 2 × 2 × 10 factorial design. The factors were the type of target (same or different), the position of stimuli on the screen (45° or 135° diagonal), and the SOA between the targets and the foveal mask (no noise, 0, 50, 100, 150, 200, 250, 300, 350, 400 ms).

### Participants

The participants were recruited between September 2022 and May 2023 through advertisements on social channels, and the sample was composed of students from the University of Padua and acquaintances of the experimenters. The data were collected confidentially by the experimenters and subsequently processed and analysed anonymously for the group analysis. The participants were unaware of the study hypotheses. All participants provided informed consent to participate, and the study was approved by the General Psychology Ethics Committee of the University of Padua under protocol number 4812. The study adhered to the requirements of the WMA Declaration of Helsinki–Ethical Principles for Medical Research Involving Human Subjects [25]. In our previous online experiment (Experiment 1 in Contemori et al. 2022 [8]), 56 subjects were included in the analysis, performing 528 trials each (excluding practice) for a total of 29568 trials. To achieve similar statistical power, we concluded the data collection after testing 47 participants (30 females) who met the inclusion criteria and performed 600 trials each, for a total of 28200. To ensure data reliability, we set an inclusion criterion of at least 60% accuracy. All participants met this criterion and were thus included in the study. The age range of the participants was 20 to 38 years, with a mean age of 24.28 years. All subjects had a normal or corrected-to-normal vision. The participants were contacted by email among the students of the Department of General Psychology at the University of Padua.

### Procedure

Before starting the test, to familiarize participants with the task, each subject watched a video in which the task and the stimuli were described. The task consisted of comparing the two peripheral stimuli and making a same/different judgment by pressing the "n" or "m" key on the computer keyboard. There was no time pressure and participants were instructed to respond as accurately as possible. Total duration of the experiment was around 90 minutes including introduction and debriefing. Each subject performed 600 trials subdivided in three blocks. Before starting the first experimental block, subjects completed a practice block in which feedback was given after each response. The practice consisted of 24 × 11 trials for a total of 80 trials and lasted about 4 minutes, resulting in a shorter practice than the original study. During the practice block, visual feedback was provided to inform the participant about the accuracy of their response.

The experiment was generated using PsychoPy3 [26]. Stimuli were displayed on an Eizo ColorEdge CS2420 with gamma correction, 1920 × 1200 pixel resolution, 60 Hz, and 61.1 cm diagonal size. Each participant sat in a quiet, dimly lit room, approximately 57 cm from the screen, using a chin rest. An eye tracker (Gazepoint GP3) was used to monitor fixation. The stimulus was not presented unless participants looked within 2 degrees from the fixation point.

Throughout the course of the experiment, the fixation cross was positioned at the centre of the screen. At the onset of each trial, two targets were simultaneously presented for a duration of 100 ms. The targets were located at diametrically symmetric positions within opposing quadrants of the screen and were pseudo randomly presented in either quadrants 1 and 3 or quadrants 2 and 4. This randomization was implemented to eliminate any expectation bias regarding target location. Following this, a dynamic, 7 × 7 deg coloured noise patch was presented for 83 ms at eight SOAs of 0, 50, 100, 150, 200, 250, 300, 350, and 400 ms, while the fixation cross remained in place. A baseline condition without any noise was also included. Participants were required to wait until the stimulus (target + mask) disappeared before providing a response. The response was only recorded starting from 600 ms after target onset. Therefore, at the longest SOA, the keyboard lock extended 117 ms beyond the mask disappearing. For this reason, reaction time are not analysed in this study. Given the challenging task and the instructions emphasizing accuracy rather than speed, average reaction times were longer than 600 ms. After removing outliers beyond 2.5 standard deviations, the reaction times were distributed as follows: Minimum 0.601, 1st Quartile 0.774, Median 0.921, Mean 1.078, 3rd Quartile 1.179, Maximum 3.588. There was no perceived waiting time before responding. The test was self-paced, and the next trial began 1 s after the response key was pressed.

### Stimuli

The target stimuli in this study were abstract three-dimensional (3D) shapes of the spikies category, as depicted in Fig 1. More details about this type of stimuli are available in the original study [7]. The stimuli had an average size of 3 × 1.5 degrees of visual angle and were presented at an eccentricity of 7 degrees. The 3D shapes differed on four primary characteristics, namely, the length and orientation of both upper and lower spikes, also illustrated in Fig 1.

At each trial, two shapes were randomly selected from a pool of 1296 possible shapes. The objects in each pair could be either "same" or "different." According to the factorial design, half of the trials (total = 28200, half = 14100) were labelled as "same" and the other half as "different." Due to the full randomization of images, 16 trials among the "different" category contained identical objects. These specific trials were reclassified as "same" prior to conducting further analysis. As in the original studies [7, 8], dissimilarity between shape pairs was determined by variation across multiple levels of one or more of the four manipulated features. Since the similarity between objects does not vary linearly for different combinations of these four characteristics [27], we utilized a metric to quantify the degree of similarity that closely approximates human judgment in the same/different task. For further details, refer to the "Image similarity metrics" section in the Methods.

### Image similarity metrics

Image similarity refers to the degree to which two images are similar to each other in terms of shape, colour, texture, and other visual factors. The more similar the images are, the more difficult it becomes to discriminate them. Although the features variation performed during the artificial object creation should produce gradually changing objects in terms of pixels overlapping, pixel-level similarity is not a good predictor for human similarity judgments [27]. For this reason in the recent years other similarity metrics have been developed with the intention to simulate the discrimination ability of a human observer [28]. To date it is not clear which one better captures the human perception of image similarity [29]. In the plethora of algorithms proposed, some are biologically inspired [28, 30], while others are based on computational models [29].

As an initial step in our analysis, we computed image similarity for pairs of stimuli using various algorithms. Through evaluating these metrics based on their R-squared values in regression analysis with task accuracy, we sought to identify the metric that best aligned with performance. In the subsequent analyses, this metric would be used to investigate the interaction between the masking effect and similarity. The similarity metrics examined were the Structural Similarity Index [31], the Gabor-Jet model-based similarity index [30], Haar wavelet-based perceptual similarity index [28], and the Jaccard similarity coefficient [32]. By considering these diverse similarity metrics, the aim was to capture various factors that may contribute to human perception of image similarity.

The Structural Similarity Index (SSIM) is a metric that considers structural information in addition to pixel values to measure image similarity. We calculated the index by means of the “ssim()” function in MATLAB [33]. SSIM is more effective than traditional methods that only consider pixel values because it assumes that the human visual system is highly sensitive to changes in structural information, such as edges and textures. SSI compares the luminance, contrast, and structure of two images to measure their structural similarity. To calculate the SSIM, two images are divided into small windows, and the structural similarity values of all the windows are averaged.

The Gabor-Jet model is a mathematical model used to measure the similarity between complex visual stimuli, such as images. We calculated the index by means of the MATLAB code provided by the authors https://geon.usc.edu/GJW/. It extracts features from images using a set of Gabor filters at different scales and orientations and calculates similarity by convolving an image with a bank of Gabor filters. The Gabor-Jet features capture the local frequency and orientation information of the image and are used to calculate the similarity between two images by computing the correlation between the two sets of features. Similarity was calculated by correlating the magnitude obtained by simulating complex cell responses with default parameters. The Gabor-Jet model is biologically plausible as it is inspired by the properties of the visual system in the brain [30].

The Haar wavelet-based perceptual similarity index (HaarPSI) utilizes the Haar wavelet transform, which is particularly effective in capturing abrupt changes in images. We calculated the index by means of the MATLAB code provided by the authors http://www.math.uni-bremen.de/cda/HaarPSI/. This metric focuses on perceptually relevant features, such as edges and texture, and quantifies the similarity based on the wavelet coefficients. HaarPSI has demonstrated superior correlation with human opinion scores on extensive benchmark databases compared to traditional full reference quality metrics [28].

The Jaccard similarity coefficient (also known as intersection over union) is a metric commonly used in the field of computer vision and image processing. Prior to Jaccard similarity extraction, images were converted into binary format using the “im2bw()” function in MATLAB with a threshold of 0.2. This threshold was chosen empirically to retain the main silhouette of objects intact and avoid isolated clusters of white pixels. After image binarization, we calculated the coefficient by means of the “jaccard()” function in MATLAB [33]. It measures the similarity between two sets of data by calculating the intersection over the union of the sets [32]. In the context of image similarity, the Jaccard index compares the overlapping regions between two images to determine their similarity. It provides a simple and intuitive measure that is often used as a baseline for evaluating other similarity metrics [34].

**Fig 2:**
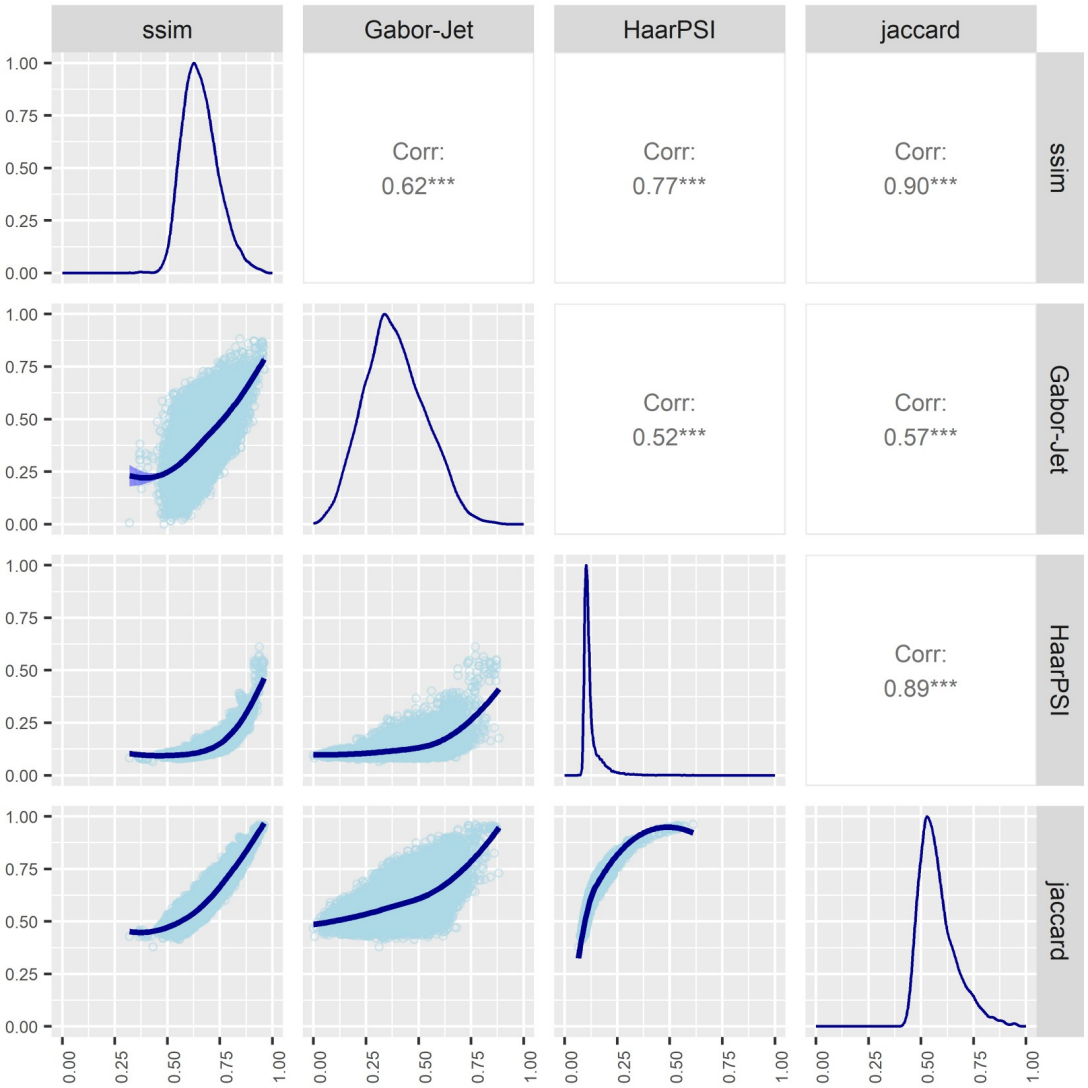
Scatter plot matrix. The matrix shows how all the possible pairs of similarity metrics were related to each other. On the top of the diagonal the value of the Pearson correlation. On the diagonal the distribution of each metric. On the bottom of the diagonal the bivariate scatter plots with LOESS smoothed fits are displayed.

As a measure of consistency between metrics, we calculated a correlation matrix. For consistency, metrics were rescaled so that a value of one indicated that the two images in the couple were identical, and zero indicated that images were totally different. Fig 2 shows the correlation matrix for the four metrics.

Despite the consistently high correlation (above 0.50 for each pair of metrics), the Jaccard coefficient had the highest average correlation with a mean R^2^ of 0.79. In this case, Jaccard similarity alone was able to approximate the information provided by the other indices effectively. To confirm that the Jaccard coefficient was the best candidate, we fitted a generalized linear mixed model for each metric after scaling, where accuracy in individual “different trials” served as the dependent variable and image similarity acted as the predictor. To account for individual variability, we also included in the model a random intercept and a random slope for each participant. Marginal R^2^ were calculated by means of the “model_performance()” function from the R package “performance” [35]. Conditional R^2^ considers the variance of both the fixed effects and the random effects. Among the metrics examined, the Jaccard similarity coefficient demonstrated the strongest predictive ability, yielding an R^2^ value of 0.169. The R^2^ values for the remaining metrics are presented in Table 1, along with the Akaike Information Criterion corrected for small sample sizes (AICc) and the delta AICc (ΔAICc).

**Table 1:** Fit of the similarity models.

Similarity Metric	AICc	ΔAICc	R^2^
Jaccard	13827.438	0	0.169
HaarPSI	14004.970	177.532	0.144
SSIM	14165.140	337.702	0.137
Gabor-Jet	14409.636	582.198	0.103

The table reports AICc (Akaike Information Criterion corrected for small sample sizes), ΔAICc (difference in AICc values), and R^2^ (coefficient of determination) calculated for a generalized linear mixed model with accuracy in the "different" trials as dependent variable and similarity between images a predictor.

**Fig 3:**
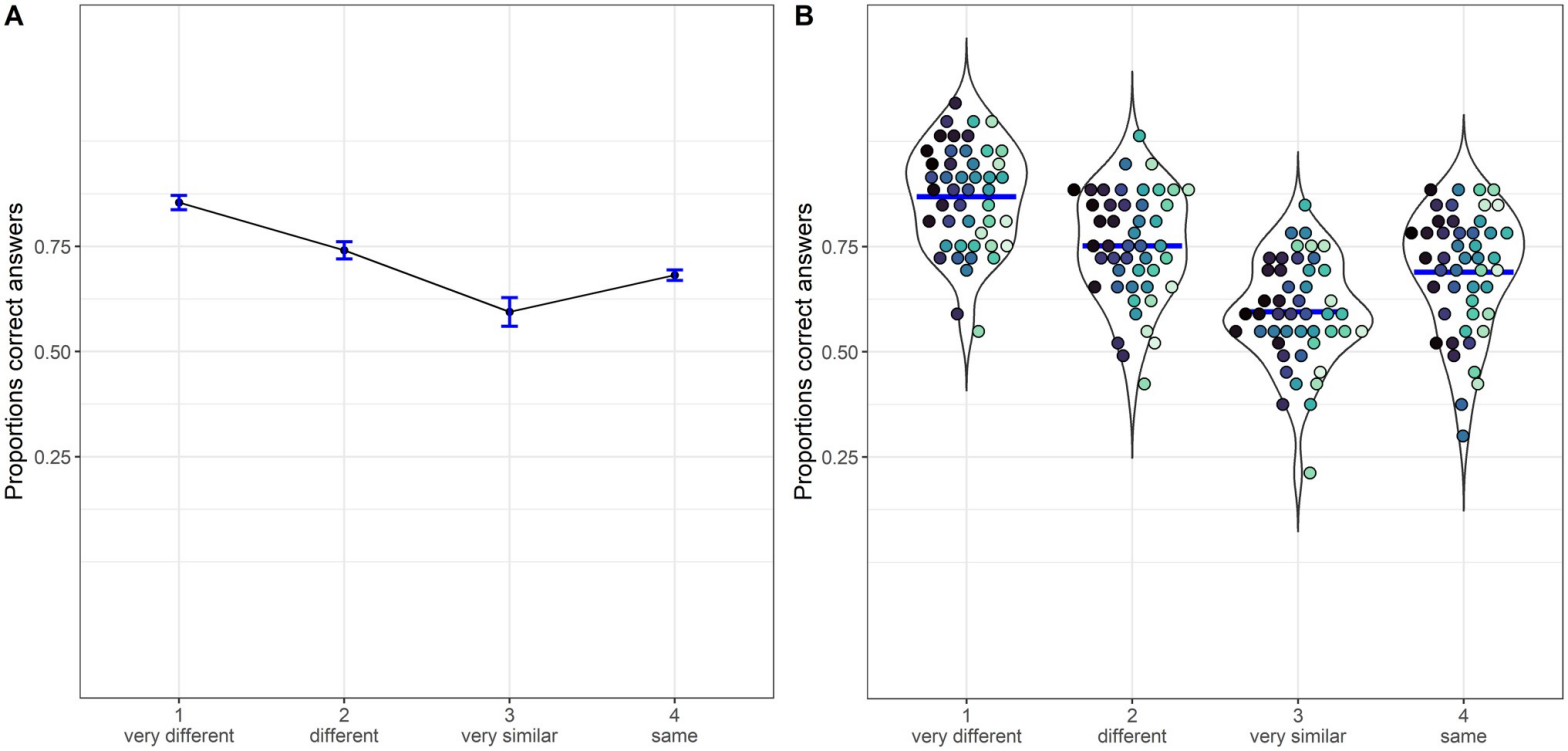
Proportions of correct answers as a function of the similarity level. Level 1 contains pairs of very different objects, and level 4 pairs of identical objects. The proportions are depicted using a non-linear scale, specifically the "asn_trans()" scale for arcsine. Panel A displays cumulative proportions, with bars representing 95% confidence intervals, adjusted using the Tryon method. These adjustments were calculated over Anscombe-transformed scores using the “superb” [37] package, and subsequently transformed back into proportions. Panel B depicts individual proportions, with each color shade corresponding to a different participant. The mean is indicated by a horizontal blue line.

After establishing that the Jaccard coefficient is the best among the selected metrics in predicting accuracy, we divided the ’different’ trials into three groups based on the similarity level of the objects. The division into groups was performed using the “discretize()” function from the “arules” [36] package in R. This function converts a numeric vector into a factor with bins based on k-means clustering. As a result, we created a similarity variable with different levels representing the degree of similarity between object pairs. Level 1 corresponds to pairs of highly different objects with an average Jaccard coefficient of 0.513 (sd = 0.031), level 2 corresponds to pairs of different objects with an average Jaccard coefficient of 0.609 (sd = 0.034), while level 3 comprises pairs of highly similar objects with an average Jaccard coefficient of 0.755 (sd = 0.061). As expected, we observed that the average accuracy decreased with increasing levels of similarity almost linearly from roof to floor performance. Specifically, the accuracy was 0.84 for level 1 (N = 7148), 0.74 for level 2 (N = 5090), and 0.60 for level 3 (N = 1846). Additionally, we had a separate level, level 4, which consisted of trials conducted under "same" conditions, yielding an average accuracy of 0.69. To visually represent this trend, Fig 3 displays the proportion of correct answers as a function of the level of similarity after the discretization process. The levels of similarity were subsequently employed as an ordered factor in the following analyses.

### Data analysis

Analysis were performed in R [38]. To analyse the mask effects at the baseline we analysed accuracy data in the peripheral same/different task by fitting a generalized linear mixed model with two factors, the presence/absence of the mask and the same/different condition. To control for the within-subjects correlation typical of repeated measures, we also included an individual random intercept and individual random slope for both the mask presence and the stimulus type in the model. Mixed models were estimated with a Restricted Maximum Likelihood procedure (REML) with the function “glmer()” from the “lme4” [39] package. Next, we tested the fixed effects using a type III Wald-test with the “Anova()” function from the “CAR” package [40].

Similarly, to analyse the effect of the mask–SOA, we analysed accuracy data by fitting a model with the SOA as ordered factor (8 levels), and the same/different condition. We also included an individual random intercept and individual random slope for the same/different conditions in the model. After testing the fixed effects using a type III Wald-test, we tested orthogonal contrasts between SOA to verify the presence of a dip/peak in performance for the “same” and “different” conditions separately. 95% confidence intervals were adjusted with Bonferroni correction for 6 estimates. P values for the z test were adjusted with False Discovery Rate (FDR) method for 6 tests. The quadratic contrast can be considered a test of whether a quadratic term could be included given that a linear term is already in the model. Hence, it serves as a hierarchical test of a quadratic model (with both linear and quadratic terms) versus a linear model. Then, to assess the location of the dip/peak, we compared each level of SOA with the baseline no-noise condition separately for the “same” and “different” conditions by means of 18 contrasts. 95% confidence intervals were adjusted with Bonferroni correction for 18 estimates. P values for the z test were adjusted with FDR method for 18 tests. We also compared the same/different conditions within each SOA.

Lastly, similar analytical approach was applied to the study the effect of similarity. In this case, the factors in the model were the SOA (8 levels, ordered), and the similarity (4 levels, ordered). We also included an individual random intercept for the participant in the model.

For all the models in this study, we performed model assumption checks using the “DHARMa” [41] R package. This package employs a simulation-based approach to analyze residuals for fitted Generalized Linear Mixed Models (GLMMs). The analysis indicated that none of the models exhibited overdispersion, underdispersion, or heteroscedasticity.

## Results

All participants included in the analyses performed above chance level and below the ceiling effect. The minimum accuracy observed was 0.60, while the maximum accuracy reached 0.86. In the following sections are the results of the generalized mixed models for accuracy in the baseline condition (no mask, 0 ms), in the “same” vs “different”, and in the interaction with the similarity based on the discretized Jaccard coefficient.

### Mask effects: In the “same” vs “different” conditions

#### Baseline

**Fig 4:**
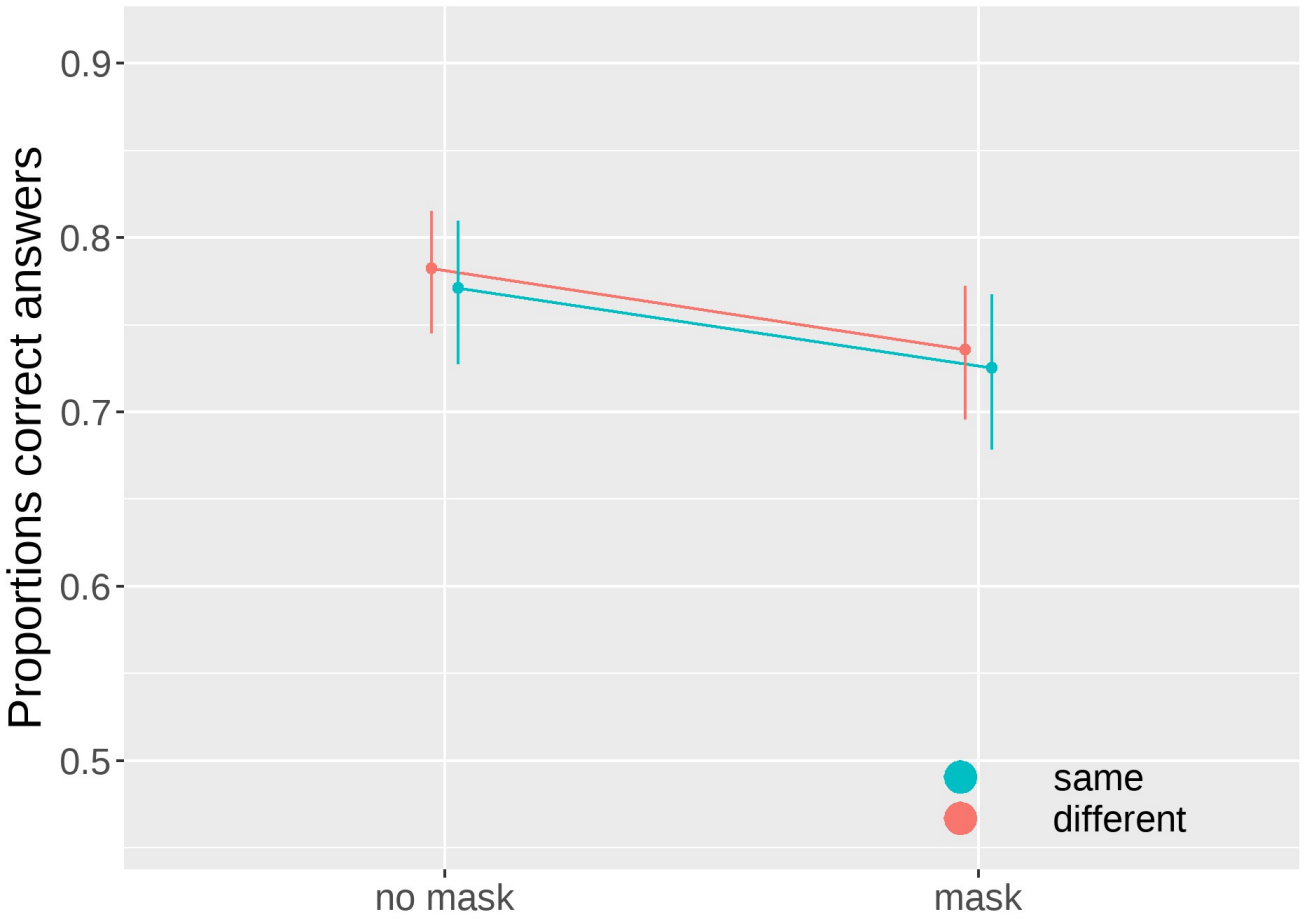
Proportions of correct answers as a function of the presence of mask at the onset of the stimulus (SOA = 0) for the “same” and “different” conditions. Bars represent 95% confidence intervals.

We compared the control condition without a mask and the condition with the mask presented simultaneously with the stimulus, separately for the "same" and "different" conditions. The results of the Type III Wald chi-square tests indicate a significant effect of mask (W(1) = 13.7489, p< 0.001), but no difference between conditions (W(1) = 0.121, p = 0.728), and not interaction (W(1) = 0.007, p = 0.932), as shown in Fig 4. The inclusion of the mask led to a decline in performance of approximately 5%. Notably, this performance decline was present for "same" and "different" conditions, suggesting that the overall number of "different" responses remained unchanged (i.e., no criterion shift occurred).

#### SOA effect

**Fig 5:**
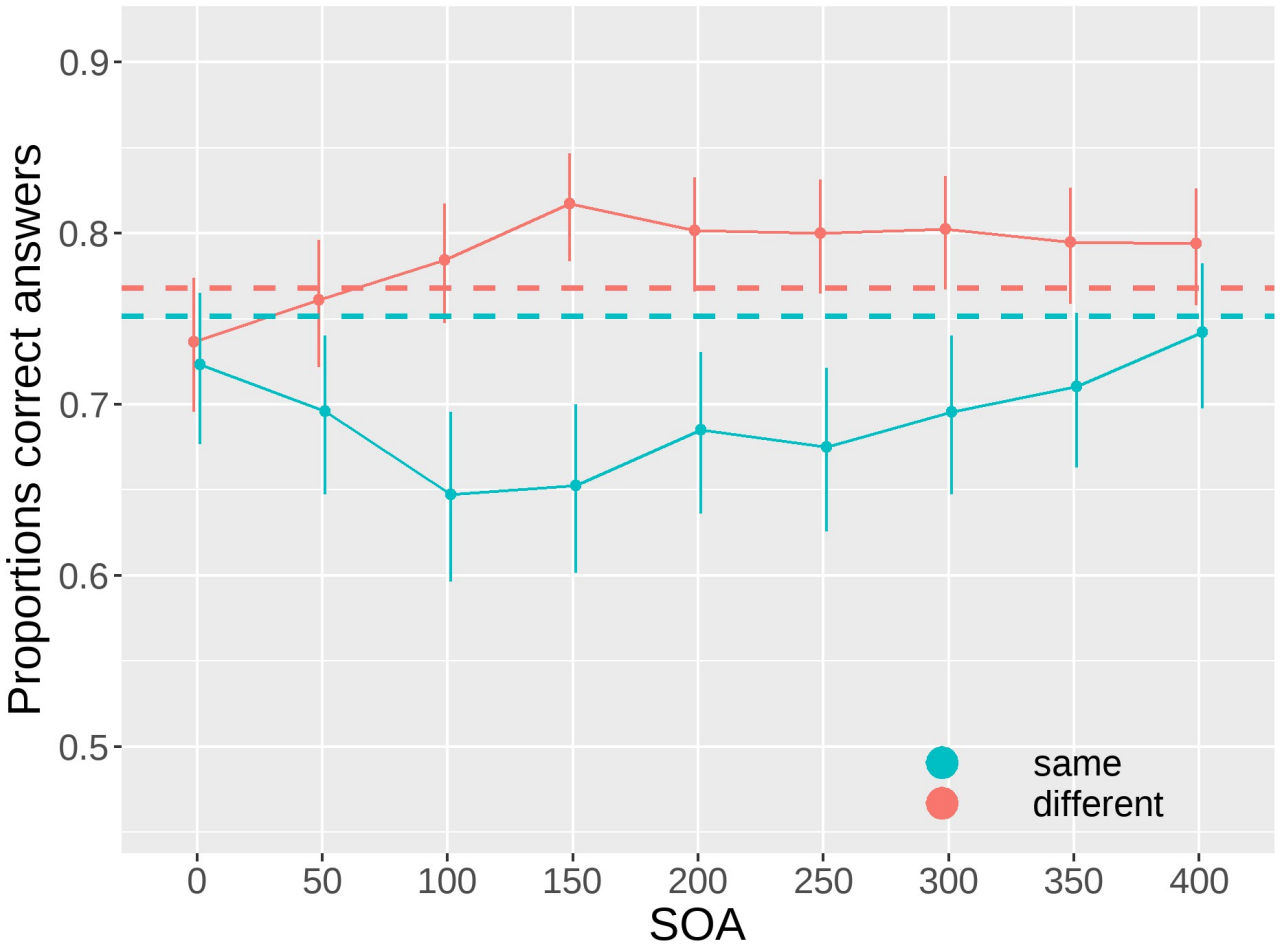
Proportions of correct answers as a function of the onset asynchrony of the mask relative to the stimulus (SOA) for the “same” and “different” conditions. Dashed lines represent the no mask baseline performance for the “same” (cyan) and “different” (red) conditions. Bars represent 95% confidence intervals.

We present the results of the effect of varying the SOA, separately for the "same" and "different" conditions. The results of the Type III Wald chi-square tests indicate a significant effect of SOA (W(8) = 24.988, p = 0.002), a significant difference between conditions (W(1) = 10.827, p = 0.001), and a significant interaction (W(8) = 60.736, p< 0.001), as shown in Fig 5.

The mask at different SOAs led to an opposite variation in performance in the “same” and “different” conditions. With performance increasing in the “different” and decreasing in the “same”. Notably, this dissociation suggests that the overall number of "different" responses increased (i.e., criterion shift occurred). Moreover, orthogonal polynomial contrasts show significant linear and quadratic effect for the SOA in both the “same” (linear: z-score(inf) = 2.411, p = 0.048; quadratic: z-score(inf) = 5.775, p< 0.001) and “different” (linear: z-score(inf) = 3.969, p< 0.001; quadratic: z-score(inf) = -4.168, p< 0.001) conditions suggesting that the effect of mask was not linear over time (Table 2).

**Table 2:** Orthogonal contrasts for the SOA in the “same” and “different” conditions.

contrast	condition	estimate	SE	df	asymp.LCL	asymp.UCL	z.ratio	p.value
**linear**	different	1.981	0.499	Inf	0.664	3.298	3.969	<0.001***
**quadratic**	different	-14.301	3.431	Inf	-23.354	-5.248	-4.168	<0.001***
**cubic**	different	2.710	2.053	Inf	-2.706	8.125	1.320	0.374
**quartic**	different	2.038	2.917	Inf	-5.656	9.733	0.699	0.582
**degree 5**	different	-1.432	1.421	Inf	-5.179	2.316	-1.008	0.470
**degree 6**	different	1.063	2.923	Inf	-6.647	8.774	0.364	0.716
**linear**	same	1.136	0.471	Inf	-0.107	2.378	2.411	0.048*
**quadratic**	same	18.424	3.190	Inf	10.007	26.840	5.775	<0.001***
**cubic**	same	-2.879	1.892	Inf	-7.870	2.111	-1.522	0.213
**quartic**	same	2.506	2.689	Inf	-4.588	9.599	0.932	0.351
**degree 5**	same	1.423	1.291	Inf	-1.983	4.828	1.102	0.324
**degree 6**	same	-3.885	2.645	Inf	-10.863	3.094	-1.469	0.213

The table reports orthogonal contrasts for the SOA in the “same” and “different” conditions. Results are given on the log odds ratio (not the response) scale. Confidence level used: 0.95. Conf-level adjustment: Bonferroni method for 6 estimates. P value adjustment: FDR method for 6 tests.

Results for the contrasts against the baseline (no mask) are in Table 3. Results show a significant difference for the "same" condition at 50 ms (z-score(inf) = -2.470, p = 0.035), 100 ms (z-score(inf) = -4.474, p< 0.001), 150 ms (z-score(inf) = -4.271, p< 0.001), and 200 ms (z-score(inf) = -2.931, p = 0.015), 250 ms (z-score(inf) = -3.341, p = 0.005), and 300 ms (z-score(inf) = -2.486, p = 0.035). Among the significant contrasts for the “same” condition the dip in performance (low accuracy) was found at 100 ms, with an estimate of 0.647. Concerning the "different" condition we found only a significant difference from the baseline at 150 ms (z-score(inf) = 2.850, p = 0.016), with an estimate peak in performance of 0.817. It is also important to notice that when comparing the two conditions by SOA, they differed significantly at 100 ms (z-score(inf) = 3.932, p< 0.001), 150 ms(z-score(inf) = 4.946, p< 0.001), 200 ms (z-score(inf) = 3.531, p< 0.001), 250 ms (z-score(inf) = 3.741, p< 0.001), 300ms (z-score(inf) = 3.280, p = 0.0010) and 350 (z-score(inf) = 2.603, p = 0.0092), but not at 0 ms (z-score(inf) = 0.393, p = 0.6944), 50 ms (z-score(inf) = 1.894, p = 0.0582), and 400 ms (z-score(inf) = 1.662, p = 0.0964).

**Table 3:** Contrasts for each SOA level against the baseline no mask for the “same” and “different” conditions.

SOA	condition	proportion	SE	df	asymp.LCL	asymp.UCL	null	z.ratio	p.value
**0**	different	0.737	0.020	Inf	0.672	0.792	0.7667411	-1.557	0.169
**50**	different	0.761	0.019	Inf	0.700	0.813	0.7667411	-0.312	0.755
**100**	different	0.784	0.018	Inf	0.726	0.833	0.7667411	0.965	0.376
**150**	different	0.817	0.016	Inf	0.764	0.861	0.7667411	2.850	0.016*
**200**	different	0.801	0.017	Inf	0.746	0.848	0.7667411	1.924	0.107
**250**	different	0.800	0.017	Inf	0.744	0.846	0.7667411	1.842	0.107
**300**	different	0.802	0.017	Inf	0.747	0.848	0.7667411	1.978	0.107
**350**	different	0.795	0.017	Inf	0.738	0.842	0.7667411	1.535	0.169
**400**	different	0.794	0.017	Inf	0.737	0.841	0.7667411	1.508	0.169
**0**	same	0.723	0.023	Inf	0.651	0.786	0.7511958	-1.280	0.241
**50**	same	0.696	0.024	Inf	0.621	0.762	0.7511958	-2.470	0.035*
**100**	same	0.647	0.025	Inf	0.568	0.719	0.7511958	-4.474	<0.001***
**150**	same	0.652	0.025	Inf	0.574	0.724	0.7511958	-4.271	<0.001***
**200**	same	0.685	0.024	Inf	0.609	0.752	0.7511958	-2.931	0.015*
**250**	same	0.675	0.024	Inf	0.598	0.744	0.7511958	-3.341	0.005**
**300**	same	0.696	0.024	Inf	0.620	0.762	0.7511958	-2.486	0.035*
**350**	same	0.710	0.023	Inf	0.637	0.774	0.7511958	-1.849	0.107
**400**	same	0.742	0.022	Inf	0.672	0.802	0.7511958	-0.415	0.718

Results are given on the response scale (proportions of correct answers). The null hypothesis (null) is the proportion of correct answers in the no mask baseline conditions. Confidence level used: 0.95. Conf-level adjustment: Bonferroni method for 18 estimates. Intervals are back-transformed from the logit scale. P value adjustment: FDR method for 18 tests. Tests are performed on the logit scale. The highest proportion of correct answers for the “different” (SOA = 150 ms, prop = 0.817) and the lowest proportion of correct answers for the “same” conditions (SOA = 100 ms, prop = 0.647) are underscored in the table.

### Mask effects: Interaction with the similarity

**Fig 6:**
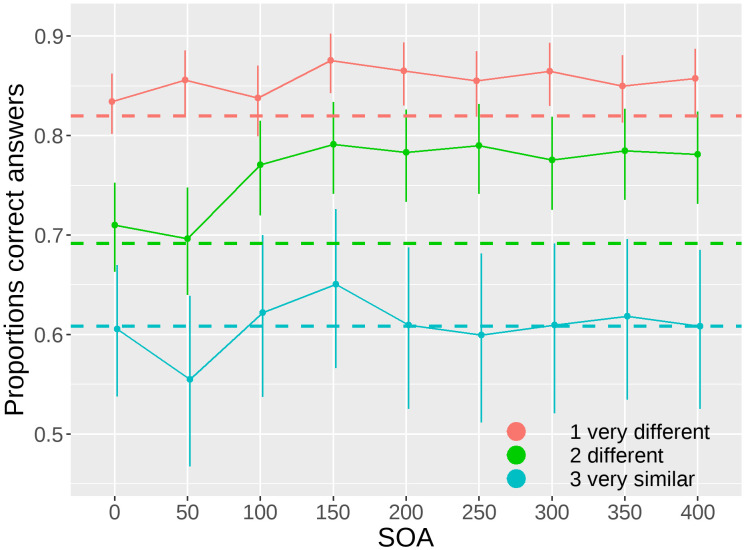
Proportions of correct answers as a function of the onset asynchrony of the mask relative to the stimulus (SOA) and the similarity level. Dashed lines represent the no mask baseline performance for each of the 3 similarity levels, 1 “very different” (red), 2 “different” (green), 3 “very similar” (cyan). Bars represent 95% confidence intervals.

We present the results of the varying SOA as a function of object similarity. The results of the Type III Wald chi-square tests indicate a significant effect of SOA (W(8) = 23.083, p = 0.003), a significant effect of similarity (W(3) = 490.417, p< 0.001), and a non-significant interaction (W(24) = 15.785, p = 0.468), as shown in Fig 6.

Orthogonal polynomial contrasts show significant linear and quadratic trends for SOA in the second level of similarity (linear: z-score(inf) = 3.920, p< 0.001; quadratic: z-score(inf) = -2.843, p = 0.013), suggesting that the effect of mask was not linear over time. For the other two similarity levels there was no significant trend (Table 4).

**Table 4:** Orthogonal contrasts for the SOA in the “same” and “different” conditions.

contrast	similarity	estimate	SE	df	asymp.LCL	asymp.UCL	z.ratio	p.value
**linear**	1	0.796	0.753	Inf	-1.189	2.782	1.058	0.716
**quadratic**	1	-8.448	5.146	Inf	-22.023	5.128	-1.642	0.604
**cubic**	1	1.706	3.119	Inf	-6.522	9.934	0.547	0.716
**quartic**	1	2.423	4.581	Inf	-9.664	14.510	0.529	0.716
**degree 5**	1	0.570	2.252	Inf	-5.372	6.512	0.253	0.800
**degree 6**	1	-2.942	4.642	Inf	-15.188	9.304	-0.634	0.716
**linear**	2	2.961	0.756	Inf	0.968	4.955	3.920	<0.001***
**quadratic**	2	-14.848	5.222	Inf	-28.625	-1.071	-2.843	0.013*
**cubic**	2	1.719	3.176	Inf	-6.661	10.099	0.541	0.588
**quartic**	2	5.834	4.604	Inf	-6.311	17.980	1.267	0.308
**degree 5**	2	-3.607	2.277	Inf	-9.614	2.400	-1.584	0.226
**degree 6**	2	3.685	4.707	Inf	-8.733	16.103	0.783	0.520
**linear**	3	0.508	1.116	Inf	-2.436	3.452	0.455	0.779
**quadratic**	3	-4.614	7.549	Inf	-24.531	15.303	-0.611	0.779
**cubic**	3	0.963	4.683	Inf	-11.390	13.317	0.206	0.837
**quartic**	3	4.236	6.765	Inf	-13.613	22.085	0.626	0.779
**degree 5**	3	-5.017	3.362	Inf	-13.887	3.852	-1.492	0.779
**degree 6**	3	4.402	6.950	Inf	-13.933	22.737	0.633	0.779

The table reports orthogonal contrasts for the SOA for each of the 3 similarity levels, 1 “very different”, 2 “different”, 3 “very similar”. Results are given on the log odds ratio (not the response) scale. Confidence level used: 0.95. Conf-level adjustment: Bonferroni method for 6 estimates. P value adjustment: FDR method for 6 tests.

Results for the contrasts against the baseline (no mask) for each similarity level are reported in Table 5. Results show a significant difference for level 1 “very different” at 150 ms (z-score(inf) = 3.131, p = 0.007), 200 ms (z-score(inf) = 2.534, p = 0.034), 300 ms (z-score(inf) = 2.471, p = 0.036). For level 2 “different” at 100 ms (z-score(inf) = 2.949, p = 0.011), 150 ms (z-score(inf) = 3.691, p = 0.003), 200 ms (z-score(inf) = 3.388, p = 0.004), 250 ms (z-score(inf) = 3.727, p = 0.003), 300 ms (z-score(inf) = 3.152, p = 0.007), 350 ms (z-score(inf) = 3.512, p = 0.004), 400 ms (z-score(inf) = 3.362, p = 0.004). For level 3 “very similar” there was no significant difference at any of the SOAs.

**Table 5:** Contrasts for each SOA level against the baseline no mask for the “same” and “different” conditions.

SOA	condition	proportion	SE	df	asymp.LCL	asymp.UCL	null	z.ratio	p.value
**0**	1	0.834	0.016	Inf	0.780	0.877	0.820	0.904	0.549
**50**	1	0.857	0.017	Inf	0.797	0.901	0.820	2.012	0.100
**100**	1	0.838	0.018	Inf	0.773	0.887	0.820	0.952	0.542
**150**	1	0.876	0.015	Inf	0.820	0.916	0.820	3.131	0.007**
**200**	1	0.866	0.016	Inf	0.808	0.909	0.820	2.534	0.034*
**250**	1	0.855	0.017	Inf	0.794	0.900	0.820	1.912	0.116
**300**	1	0.865	0.016	Inf	0.806	0.907	0.820	2.471	0.036*
**350**	1	0.850	0.017	Inf	0.788	0.896	0.820	1.617	0.204
**400**	1	0.857	0.017	Inf	0.797	0.902	0.820	2.029	0.100
**0**	2	0.710	0.023	Inf	0.634	0.776	0.691	0.795	0.606
**50**	2	0.695	0.028	Inf	0.603	0.774	0.691	0.139	0.999
**100**	2	0.771	0.024	Inf	0.687	0.838	0.691	2.949	0.011*
**150**	2	0.791	0.024	Inf	0.709	0.855	0.691	3.691	0.003**
**200**	2	0.782	0.024	Inf	0.700	0.847	0.691	3.388	0.004**
**250**	2	0.790	0.023	Inf	0.709	0.853	0.691	3.727	0.003**
**300**	2	0.776	0.024	Inf	0.693	0.841	0.691	3.152	0.007**
**350**	2	0.785	0.023	Inf	0.703	0.849	0.691	3.512	0.004**
**400**	2	0.781	0.024	Inf	0.699	0.846	0.691	3.362	0.004**
**0**	3	0.606	0.034	Inf	0.497	0.705	0.608	-0.079	0.999
**50**	3	0.555	0.044	Inf	0.417	0.685	0.608	-1.228	0.395
**100**	3	0.622	0.042	Inf	0.486	0.741	0.608	0.328	0.999
**150**	3	0.650	0.041	Inf	0.514	0.766	0.608	0.999	0.537
**200**	3	0.609	0.042	Inf	0.475	0.729	0.608	0.028	0.999
**250**	3	0.599	0.044	Inf	0.459	0.725	0.608	-0.202	0.999
**300**	3	0.609	0.044	Inf	0.467	0.735	0.608	0.028	0.999
**350**	3	0.618	0.042	Inf	0.484	0.737	0.608	0.244	0.999
**400**	3	0.608	0.041	Inf	0.476	0.726	0.608	-0.002	0.999

Results are given on the response scale (proportions of correct answers). The null hypothesis (null) is the proportion of correct answers in the no mask baseline conditions. Confidence level used: 0.95. Conf-level adjustment: Bonferroni method for 27 estimates. Intervals are back-transformed from the logit scale. P value adjustment: FDR method for 27 tests. Tests are performed on the logit scale. The highest proportion of correct answers for 1 “very different” (SOA = 150 ms, prop = 0.876), 2 “different” (SOA = 150 ms, prop = 0.791), 3 “very similar” (SOA = 150 ms, prop = 0.650) are underscored in the table.

## Discussion

The present study investigates the impact of the simultaneous arrival of feedback from peripheral targets and mask information in the foveal cortex on visual perception. By manipulating the timing and content of these stimuli, we gained insights into the mechanisms underlying neural contamination and its influence on perceptual judgments. Our findings shed light on the complex interplay between different stages of visual processing and highlight the active role of the foveal cortex in shaping perception.

Consistent with previous research, our results demonstrated that the timing of stimulus presentation significantly influenced participants’ judgments of similarity. Specifically, we observed a significant decrease in performance when the two peripheral objects were identical and a significant increase in performance when the two peripheral objects were different. This finding suggests that the simultaneous arrival of feedback and mask information in the foveal cortex can lead individuals to perceive dissimilarity even when the objects are identical. The result of this perceptual bias is an overall increment in the number of “different” responses. Moreover, the timing at which the mask was more effective differed for the “same” and “different” conditions. When the two objects were identical, the dip in performance occurred at approximately 100 ms, while when the two objects were different, the peak in performance was observed around 150 ms. This delayed effect might indicate that the processing of dissimilar objects requires additional time for the foveal cortex to disentangle their features and accurately judge their similarity.

The observed increase in "different" responses aligns with the criterion shift previously reported by Contemori et al. (2022) [8] and indicates the presence of contextual contamination in the neural representation of the stimuli, where the processing of mask information influences the perception of the target objects. This finding is in line with previous research demonstrating that early visual processing stages are susceptible to interference from other retinotopic locations, leading to biased perception [6]. Furthermore, the contamination of targets’ neural representations caused by the appearance of the mask has been previously reported [6, 9, 11], suggesting that the mask is more effective when it shares task-related properties with the target stimuli [10]. This pooling of features between peripheral targets and foveal masks supports a possible link with the crowding phenomenon.

The concept of crowding is relevant to our study because it shares similarities with the observed neural contamination. Both phenomena involve the interference or contamination of neural representations due to the presence of surrounding stimuli. In crowding, the interference occurs in the periphery, where the close proximity of flankers hinders the accurate perception of the target object [19]. Similarly, in our study, we found that the simultaneous presentation of feedback and mask information in the foveal cortex influenced the perception of the target objects, leading to biased judgments of similarity.

While crowding is conventionally associated with spatial interference, our study suggests that neural contamination can also occur in the foveal cortex, where the spatial arrangement of stimuli is not a factor. The observed temporal discrepancy between sensitivity and criterion, together with the behavioural relevance of foveal feedback, cannot be fully explained by a predictive mechanism solely based on foveation. Instead, it may be more appropriate to consider the foveal retinotopic cortex as a visual sketchpad for the maintenance and manipulation of task-relevant information, akin to Baddeley’s visuospatial sketchpad (VSSP) [4]. In this context, the pooling of information, instead of occurring based on retinotopic proximity, could occur based on spatial and temporal proximity within the neural space of the VSSP.

Contrary to our expectations, we did not observe a modulation of task difficulty on the effect of the mask in our study. Regardless of the level of similarity between the two objects, we consistently observed a peak in performance in the "different" condition at approximately 150 ms. However, we found a quadratic effect of the mask-stimulus onset asynchrony only for the intermediate similarity level. It is possible that the lowest and highest similarity levels approached the performance ceiling and floor, respectively, thereby diminishing the detectable effect of SOA.

In contrast to the findings reported by Fan et al. (2016) [7] in the context of the mental rotation task, our study did not reveal a shift of the mask effect towards larger SOAs with increasing similarity of the objects. This suggests that it is not the task difficulty per se that influences the timing of foveal feedback, but rather the complexity of the mental operations performed on the targets. This finding implies that the level of cognitive load imposed on the visuospatial sketchpad determines the flexibility in the timing of foveal feedback.

The present findings have important implications for our understanding of visual perception and the role of the foveal cortex in shaping our subjective experiences. The observed neural contamination suggests that the foveal cortex does not process visual information in isolation but rather integrates feedback and mask information, potentially leading to perceptual biases. These findings challenge the traditional view of the foveal cortex as a passive receiver of information, highlighting its active role in shaping perception.

## Conclusion

In summary, our study reveals the active role of the foveal cortex in integrating feedback and mask information during visual perception. The timing of mask presentation significantly influenced judgments, while task difficulty did not modulate the masking effect. Participants consistently showed a dip in performance for the “same” condition at about 100 ms, and a peak in performance for the "different" condition at about 150 ms, independent of similarity level. Our findings are not consistent with the view of the foveal cortex as a passive receiver, highlighting its active involvement in shaping perception. Neural contamination in the foveal cortex might arise from a pooling of information between foveal and peripheral processing. In contrast to mental rotation studies, our results did not show a progressive shift in the timing of the masking effect with increasing similarity. Therefore, the timing of foveal feedback is likely regulated by the complexity of mental operations to perform and not simply by increasing task difficulty. This study advances our understanding of visual perception and the dynamic interplay between peripheral-to-foveal feedback, mask disruption, and discrimination performance.
